# Abnormal Low-Frequency Oscillations Reflect Abnormal Eye Movement and Stereovision in Patients With Comitant Exotropia

**DOI:** 10.3389/fnhum.2021.754234

**Published:** 2021-10-08

**Authors:** Juan Chen, Han Jin, Yu-Lin Zhong, Xin Huang

**Affiliations:** Department of Ophthalmology, Jiangxi Provincial People's Hospital Affiliated to Nanchang University, Nanchang, China

**Keywords:** concomitant exotropia, ALFF, fALFF, functional magnetic resonance imaging, support vector machine, machine learning

## Abstract

**Background:** Patients with comitant exotropia (CE) are accompanied by abnormal eye movements and stereovision. However, the neurophysiological mechanism of impaired eye movements and stereovision in patient with CE is still unclear.

**Purpose:** The purpose of this study is to investigate spontaneous neural activity changes in patients with CE using the amplitude of low-frequency fluctuation (ALFF) method and the machine learning method.

**Materials and Methods:** A total of 21 patients with CE and 21 healthy controls (HCs) underwent resting-state magnetic resonance imaging scans. The ALFF and fractional amplitude of low-frequency fluctuation (fALFF) values were chosen as classification features using a machine learning method.

**Results:** Compared with the HC group, patients with CE had significantly decreased ALFF values in the right angular (ANG)/middle occipital gyrus (MOG)/middle temporal gyrus (MTG) and bilateral supplementary motor area (SMA)/precentral gyrus (PreCG). Meanwhile, patients with CE showed significantly increased fALFF values in the left putamen (PUT) and decreased fALFF values in the right ANG/MOG. Moreover, patients with CE showed a decreased functional connectivity (FC) between the right ANG/MOG/MTG and the bilateral calcarine (CAL)/lingual (LING) and increased FC between the left PUT and the bilateral cerebellum 8/9 (CER 8/9). The support vector machine (SVM) classification reaches a total accuracy of 93 and 90% and the area under the curve (AUC) of 0.93 and 0.90 based on ALFF and fALFF values, respectively.

**Conclusion:** Our result highlights that patients with CE had abnormal brain neural activities including MOG and supplementary motor area/PreCG, which might reflect the neural mechanism of eye movements and stereovision dysfunction in patients with CE. Moreover, ALFF and fALFF could be sensitive biomarkers for distinguishing patients with CE from HCs.

## Introduction

Comitant exotropia (CE) is a common eye movement disorder, characterized by ocular deviation and impaired stereovision function. The prevalence of exotropia is 1.0% of all children (Govindan et al., 2005). There are several risk factors for the occurrence of strabismus including genetics (Maconachie et al., 2013), amblyopia (Shapira et al., 2018), and refractive error (Zhu et al., 2015). At present, strabismus surgery is an effective treatment for patients with strabismus. However, stereovision was not established in some patients with exotropia after surgery (Sturm et al., 2011). Thus, the pathological neural mechanism of impaired stereovision in patients with CE is still poorly understood.

Binocular vision is common to humans. Binocular processing primarily emerges when neurons receiving input from the two eyes converge onto common cells in the primary visual cortex (Basgoze et al., 2018). Binocular vision plays an important role in stereoscopic vision. Moreover, Hou et al. (2020) reported that binocular interactions share a common gain control mechanism in the striate and extra-striate cortex. Patients with strabismus with impaired stereovision were associated with brain activity changes. Chen and Tarczy-Hornoch (2011) found that the patients with strabismus had decreased cortical activity in the primary visual cortex. Meanwhile, Shi et al. (2019) demonstrated that patients with constant exotropia had higher regional homogeneity (ReHo) values in the second visual cortex. Yan et al. (2019) demonstrated that strabismus showed increased functional connectivity (FC) between the primary visual cortex and the frontal eye field. In addition, patients with strabismus are also accompanied by cerebral structural changes. Yan et al. (2010) found that patients with CE showed lower fractional anisotropy values in the middle occipital gyrus (MOG) and supramarginal gyrus. However, these existing studies were mainly focused on the visual cortex changes in the strabismus. Whether whole spontaneous brain activity changes in patients with CE is still poorly understood.

The low-frequency oscillations (LFO) (0.01–0.08 Hz) on blood oxygen level-dependent (BOLD) imaging play an important role in various neurophysiological activities including vision (Chan et al., 2017) and cognition (van Kerkoerle et al., 2014). The amplitude of low-frequency fluctuation (ALFF) is a reliable and sensitive functional MRI (fMRI) method to quantify the total power of LFO within a specific frequency range (Zou et al., 2008). In contrast to the task MRI method, this technique does not require the participants to perform any task during scanning.

Thus, this study aimed to determine whether patients with CE were associated with intrinsic brain activity dysfunction using ALFF and fALFF methods. Meanwhile, the seed-based FC method was applied to calculate time series correlation based on different ALFF and fALFF regions. Moreover, machine learning techniques (e.g., support vector machine, SVM) were applied to assess whether ALFF and fALFF values could be sensitivity biomarkers to distinguish patients with CE from healthy controls (HCs). Our findings might reflect neural mechanisms of eye movement disorder and impaired stereovision in patients with CE.

## Materials and Methods

### Participants

A total of 21 patients (15 males/6 females, mean age: 15.80 ± 2.46 years) with CE and 21 age- and sex-matched HCs (15 males/6 females, mean age: 16.00 ± 2.68 years) were recruited from Jiangxi Provincial People's Hospital Affiliated to Nanchang University. The diagnostic criteria of patients with CE were as follows: (1) CE, exodeviation angles between 15Δ and 80Δ, and (2) without a history of strabismus surgery.

The exclusion criteria of individuals with CE in this study were as follows: (1) additional ocular-related complications (e.g., cataract, glaucoma, high myopia, or optic neuritis), (2) sensory exotropia and fixed exotropia, and (3) comitant exotropia were associated with amblyopia.

All HCs met the following criteria: (1) no ocular diseases (e.g., myopia, cataracts, glaucoma, optic neuritis, or retinal degeneration), (2) binocular visual acuity ≥ 1.0, and (3) no ocular surgical history.

### MRI Acquisition

MRI scanning was performed on a 3-Tesla magnetic resonance scanner (Discovery MR 750W system; GE Healthcare, Milwaukee, WI, USA) with an eight-channel head coil with the following parameters: repetition time/echo time (TR/TE) = 2,000/25 ms, thickness = 3.0 mm, gap = 1.2 mm, acquisition matrix = 64 × 64, flip angle = 90°, field of view = 240 mm^2^ × 240 mm^2^, voxel size = 3.6 mm^3^ × 3.6 mm^3^ × 3.6 mm^3^, and 35 axial slices. All subjects underwent MRI scanning with their eyes closed without falling asleep.

### fMRI Data Processing

All preprocessing was performed using the toolbox for Data Processing and Analysis of Brain Imaging (DPABI, http://www.rfmri.org/dpabi) (Yan et al., 2016), and the following steps were followed (Yin et al., 2017): (1) remove first 10 volumes. (2) Slice timing effects and motion-corrected. (3) Individual 3D-BRAVO images were registered to the mean fMRI data, and normalized data (in Montreal Neurological Institute [MNI] 152 space) were re-sliced at a resolution of 3 mm^3^ × 3 mm^3^ × 3 mm^3^. (4) Spatial smoothing by convolution with an isotropic Gaussian kernel of 6 mm × 6 mm × 6 mm full width at half maximum. (5) Linear regression analysis was used to regress out several covariates [six head motion parameters, mean framewise displacement (FD), global brain signal, and averaged signal from white matter signal, and cerebrospinal fluid) (6). Data with linear trend were removed, and temporal band-pass was filtered (0.01–0.08 Hz). Fisher's *r*-to-*z* transformation was used to acquire an approximate normal distribution and help to reduce the impacts of individual variations on group statistical comparisons.

### ALFF and fALFF Analyses

To calculate ALFF, we converted the smoothed signal of each voxel from the time domain to frequency domain *via* fast Fourier transform (FFT) to obtain the power spectrum. The fALFF value was computed as the ratio of the power in the specific frequency band to that of the whole detected frequency range for suppressing nonspecific signals in the rs-fMRI data.

### Seed-Based Resting-State FC Analysis

To further detect the altered functional networks behind the impaired ALFF and fALFF. The regions of interest (ROIs) were selected as seeds for the whole-brain FC analysis from the significant results of fALFF and ALFF images from the comparison of patients with CE and HCs. The correlation analysis of time course was performed between the spherical seed region (6 mm) and each voxel of the whole brain for each subject.

### Ophthalmic Examination

All participants underwent several examinations including best-corrected visual acuity (BCVA), exodeviation angle, ocular motility, fusional control score, Worth 4-dot test, and Titmus stereopsis test.

### Statistical Analysis

The independent sample *t*-test was used to investigate the clinical features between the two groups.

The one-sample *t*-test was conducted to assess the group mean of zALFF and zfALFF maps. The two-sample *t*-test was used to compare the two group differences in the zALFF and zfALFF maps using the Gaussian random field (GRF) method that was used to correct for multiple comparisons and regressed covariates of age and sex and FD (two-tailed, voxel-level *P* < 0.01, GRF correction, cluster-level *P* < 0.05). The two-sample t-test was used to compare the two group differences in the FC maps.

### SVM Analysis

The SVM algorithm was performed using the Pattern Recognition for Neuroimaging Toolbox (PRoNTo) software Cyclotron Research Centre, University of Liège, Belgium (Schrouff et al., 2013). The following steps were followed: (1) the ALFF and fALFF maps served as a classification feature. (2) Then, the leave-one-out cross-validation (LOOCV) technique was used to perform SVM classifier validation. For classification, two classes were defined (patient group and HC group) and processed using a soft-margin hyper-parameter approach. The soft-margin parameters take the values 0.01, 0.1, 1, 10, 100, and 1,000 in the SVM classifier in the current version of PRoNTo, which make the model obtain the maximum interval hyperplane with the minimum classification error, and then, selected the soft-margin parameter with the lowest total error rate as the final parameter for each cycle of the cross-validation. (3) For classification, the permutation test was applied to assess the statistical significance of the total accuracy of this classification (Liu et al., 2012). The total accuracy, specificity, sensitivity, and area under the receiver operating characteristic curve (AUC) were determined to assess the classification performance of the machine learning model.

## Results

### Clinical Measurements

There were no differences in age and gender and best-corrected visual acuity between the two groups (*P* > 0.05; Table 1).

**Table 1:** Demographics and visual measurements between the two groups.

**Condition**	**CE group**	**HC group**	**T-values**	**P-values**
Gender (male/female)	(15/6)	(15/6)	N/A	N/A
Comitant category	congenital exotropia	N/A	N/A	N/A
Age (years)	15.80 ± 2.46	16.00 ± 2.68	−0.240	0.812
Handedness	21 R	21 R	N/A	N/A
BCVA-OD	1.12 ± 0.27	1.05 ± 0.23	0.899	0.374
BCVA-OS	1.18 ± 0.26	1.08 ± 0.21	1.305	0.199

*Independent t-test for the other normally distributed continuous data (means ± SD)*.

*CE, comitant exotropia; HC, healthy control*.

### ALFF and fALFF Differences

**Figure 1:**
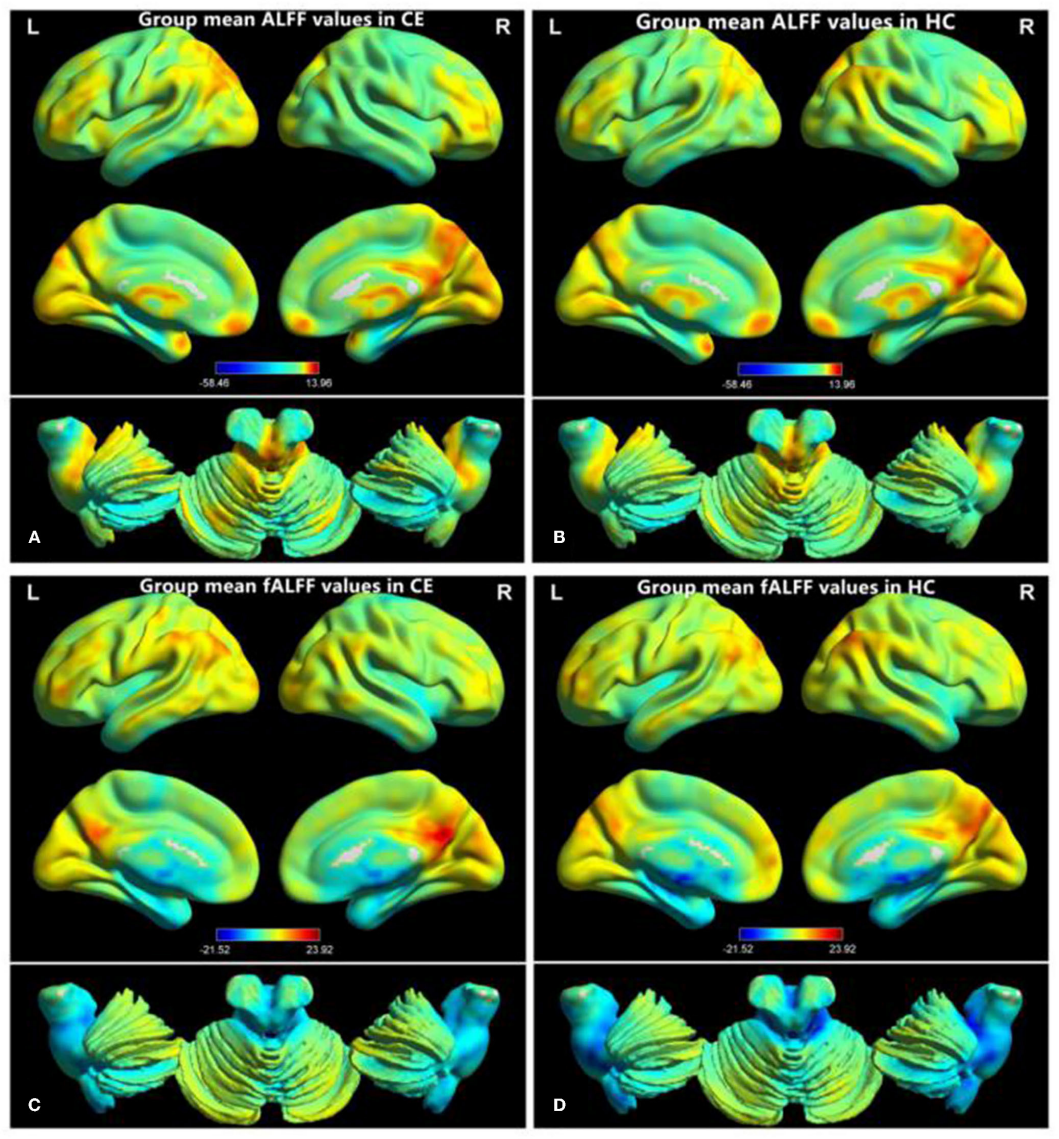
Distribution patterns of ALFF and fALFF were observed at the group level in CE and HC. Within-group mean ALFF maps within the CE **(A)** and HCs **(B)** within-group mean fALFF maps within the CE **(C)** and HCs **(D)**. ALFF, amplitude of low-frequency fluctuation; fALFF, fractional amplitude of low-frequency fluctuation; CE, comitant exotropia; HC, healthy control.

**Figure 2:**
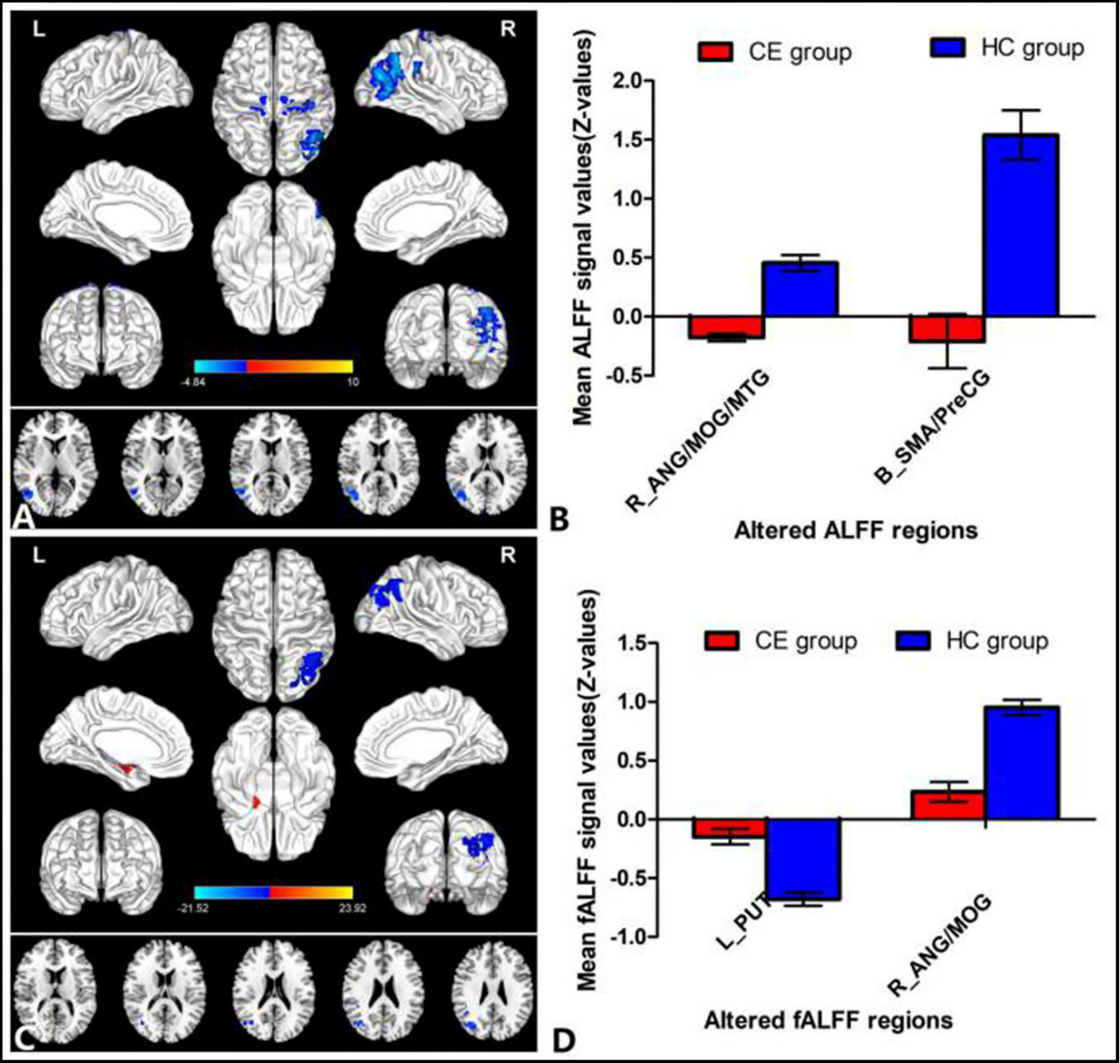
Group comparisons of the ALFF and fALFF between the CE and HCs. Significantly different ALFF was found between the two groups. **(A)** Significantly different fALFF were found between the two groups. **(C)** The blue areas denote lower ALFF and fALFF values, and the red areas denote increased ALFF and fALFF values. The mean values of altered ALFF/fALFF values are shown in a histogram **(B,D)**. ALFF, amplitude of low-frequency fluctuation; fALFF, fractional amplitude of low-frequency fluctuation; CE, comitant exotropia; HC, healthy control; ANG, angular; MOG, middle occipital gyrus; MTG, middle temporal gyrus; SMA, supplementary motor area; PreCG, precentral gyrus; PUT, putamen; R, right; L, left.

The group means of ALFF and fALFF maps of the CE and HC are shown in Figures 1A–D. Compared with the HC group, patients with CE showed significantly decreased ALFF values in the right angular (ANG)/MOG/middle temporal gyrus (MTG) and bilateral supplementary motor area (SMA)/precentral gyrus (PreCG) (Figure 2A; Table 2). Meanwhile, patients with CE showed significantly increased fALFF values in the left putamen (PUT) and decreased fALFF values in the right ANG/MOG (Figure 2C; Table 2). The mean values of altered ALFF maps were shown with a histogram (Figure 2B). The mean values of altered fALFF maps were shown with a histogram (Figure 2D).

**Table 2:** Different ALFF values between the two groups.

**Condition**	**Brain regions**	**BA**	**Peak *T*-scores**	**MNI coordinates (*x*, *y*, *z*)**	**Cluster size (voxels)**
**Different ALFF values**					
CE < HC	R_ANG/MOG/MTG	19, 40	−4.378	48, −75, 24	528
CE < HC	B_SMA/PreCG	3, 4	−4.8378	21, −24, 78	303
**Different fALFF values**					
CE>HC	L_PUT	-	4.461	−24, −9, 6	144
CE < HC	R_ANG/MOG	19, 40	−4.5814	30, −84, 33	340

*The statistical threshold was set at the voxel level with P < 0.01 for multiple comparisons using Gaussian random field theory*.

*ALFF, amplitude of low-frequency fluctuation; CE, comitant exotropia; HC, healthy control; ANG, angular; MOG, middle occipital gyrus; MTG, middle temporal gyrus; SMA, supplementary motor area; PreCG, precentral gyrus; PUT, putamen; R, right; L, left; B, bilateral*.

### FC Differences

**Figure 3:**
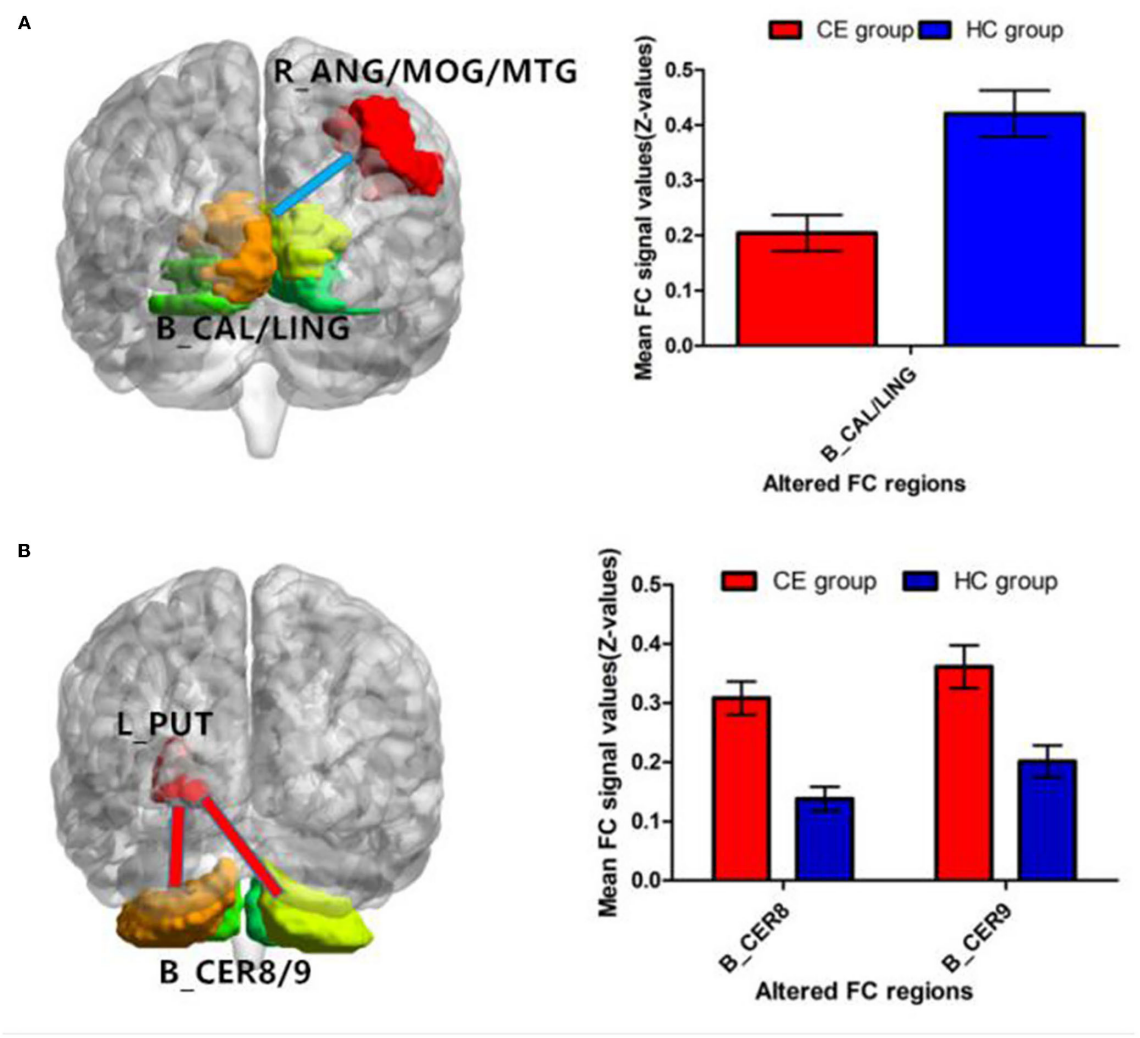
Significant z-FC map differences seeded as ROI in different ALFF/fALFF regions between the two groups. The cool color indicates decreased z-FC values. The warm color indicates increased z-FC values. Significantly different FC values seeded as ROI in R-ANG/MOG/MTG **(A)** and L-PUT **(B)**. ALFF, amplitude of low-frequency fluctuation; fALFF, fractional amplitude of low-frequency fluctuation; ROI, region of interests; FC, functional connectivity; CE, comitant exotropia; HC, healthy control; ANG, angular; MOG, middle occipital gyrus; MTG, middle temporal gyrus; CAL, calcarine; LING, lingual; PUT, putamen; CER, cerebellum.

Patients with CE showed a decreased FC between the right ANG/MOG/MTG and the bilateral calcarine (CAL)/lingual (LING) (Figure 3A; Table 3) and increased FC between the left PUT and the bilateral cerebellum 8/9 (CER 8/9) (Figure 3B; Table 3). The mean values of altered FC maps were shown with a histogram (Figures 3A,B).

**Table 3:** Different functional connectivity (FC) values between the two groups.

**Condition**	**Brain regions**	**BA**	**Peak *T*-scores**	**MNI coordinates (*x*, *y*, *z*)**	**Cluster size (voxels)**
**Seed in R-ANG/MOG/MTG**					
CE < HC	B_CAL/LING	17, 18	−4.205	−12, −102, −12	1,092
**Seed in L-PUT**					
CE>HC	B_CER8	–	4.5664	6, −63, −36	1,209
CE>HC	B_CER9	–	3.332	−33, −60, −24	91

*The statistical threshold was set at the voxel level with P < 0.01 for multiple comparisons using Gaussian random field theory*.

*ALFF, amplitude of low-frequency fluctuation; CE, comitant exotropia; HC, healthy control; ANG, angular; MOG, middle occipital gyrus; MTG, middle temporal gyrus; CAL, calcarine; LING, lingual; PUT, putamen; CER, cerebellum; R, right; L, left; B, bilateral*.

### SVM Classification Results

**Figure 4:**
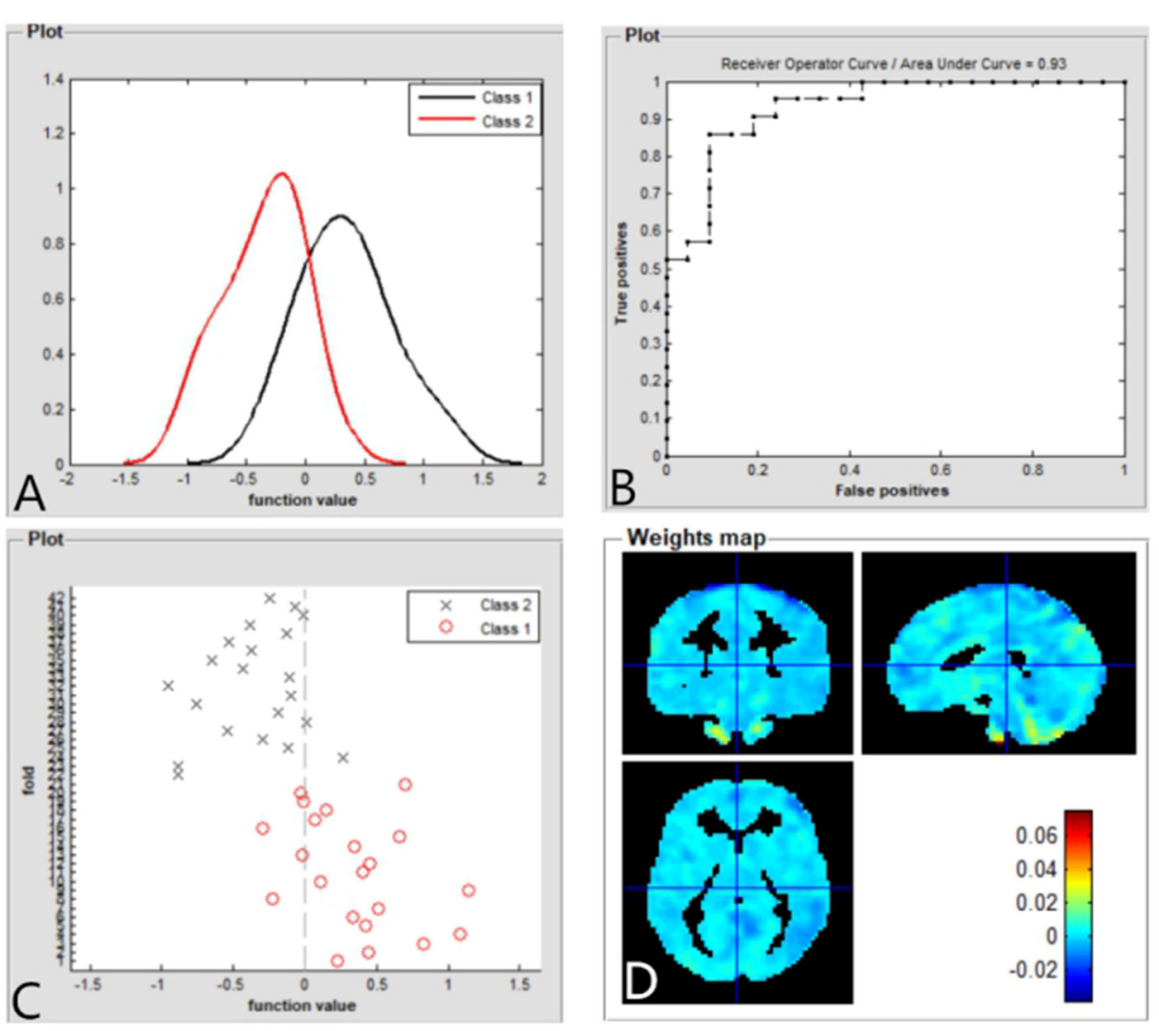
Based on ALFF values, distribution of function values of the two groups (class 1: patient group; class 2: HC group) **(A)**; the ROC curve of the classifier, and the AUC was 0.93 **(B)**; function values of the two groups (class 1: CE group; class 2: HC group) **(C)**; and weight maps for SVM models **(D)**. ALFF, amplitude of low-frequency fluctuation; ROC, receiver operator characteristic; AUC, areas under the curve; CE, comitant exotropia; HC, healthy control; SVM, support vector machine.

**Figure 5:**
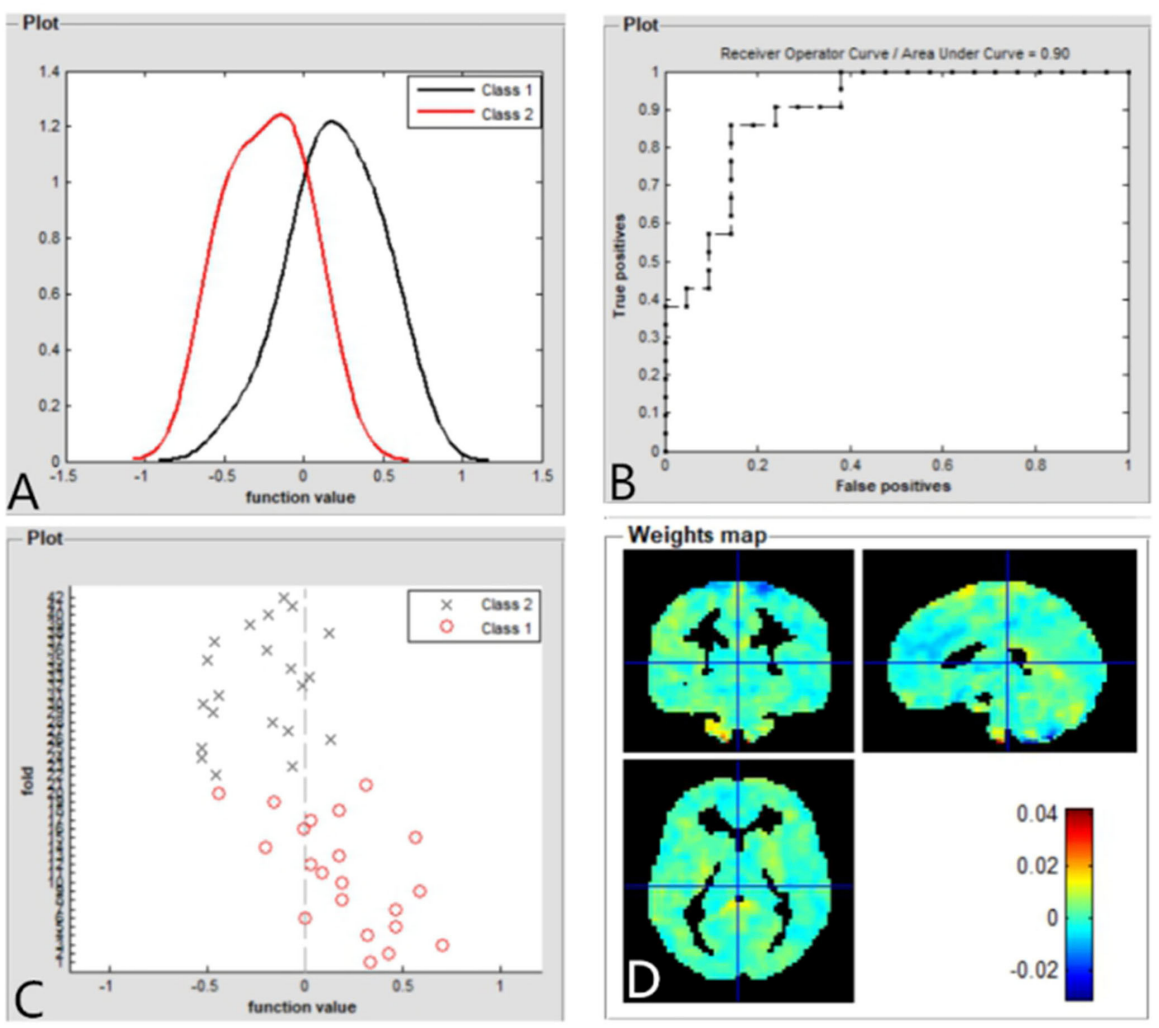
Based on fALFF values, distribution of function values of the two groups (class 1: patient group; class 2: HC group) **(A)**; the ROC curve of the classifier, and the AUC was 0.90 **(B)**; function values of the two groups (class 1: patient group; class 2: HC group) **(C)**; weight maps for SVM models **(D)**. fALFF, fractional amplitude of low-frequency fluctuation; ROC, receiver operator characteristic; AUC, areas under curve; CE, comitant exotropia; HC, healthy controls; SVM, support vector machine.

The SVM classification reaches a total accuracy of 93%. The AUC of the classification model was 0.93 based on ALFF (Figure 4). The SVM classification reaches a total accuracy of 90%. The AUC of the classification model was 0.90 based on fALFF (Figure 5).

## Discussion

The purpose of this study was to investigate the spontaneous neural activity changes in patients with CE. Compared with the HC group, patients with CE showed significantly decreased ALFF values in the right ANG/MOG/MTG and bilateral SMA/PreCG. Meanwhile, patients with CE showed significantly increased fALFF values in the left PUT and decreased fALFF values in the right ANG/MOG. Moreover, patients with CE showed a decreased FC between the right ANG/MOG/MTG and the bilateral calcarine (CAL)/lingual (LING) and increased FC between the left PUT and the bilateral CER 8/9. Furthermore, the SVM classification reaches a total accuracy of 93 and 90% and the AUC of 0.93 and 0.90 based on ALFF and fALFF values, respectively.

An important finding is that patients with CE had significantly decreased ALFF values in the bilateral SMA/PreCG. The SMA/PreCG regions are important motor control-related brain functions. SMA is involved in the saccadic task and eye movement control (O'Driscoll et al., 1995; Campos et al., 2005). In addition, the supplementary eye field is located in SMA, which plays an important role in eye movement control and goal-directed behavior (Parton et al., 2007; Stuphorn, 2015). The frontal eye field is located in PreCG. Previous studies have revealed that the PreCG plays an important role in eye movement control (Blanke et al., 2000; Amiez and Petrides, 2009). Thus, we speculated that the significantly decreased ALFF values in the SMA/PreCG regions might contribute to the eye movement disorders in patients with CE.

In addition, another interesting finding is that patients with CE showed significantly decreased ALFF and fALFF values in the right ANG/MOG/MTG. Previous studies have identified a number of visual areas responsive to disparity-defined depth (Brouwer et al., 2005). Gonzalez et al. (2005) demonstrated that the parietal-occipital-temporal junction plays an important role in the processing of stereoscopic information. Meanwhile, previous studies demonstrated that middle temporal (MT) neurons showed a stronger ability of MT neurons to signal binocular disparity in moving vs. stationary random-dot stereograms, presence of disparity-sensitive cells in MT+ and that these neurons can detect surface orientation on the basis of disparity gradients (Nguyenkim and DeAngelis, 2003; Palanca and DeAngelis, 2003). Consistent with these findings, we speculated that the significantly decreased ALFF and fALFF values in the right ANG/MOG/MTG regions might contribute to the binocular and stereoscopic vision disorders in patients with CE.

Furthermore, patients with CE showed a decreased FC between the right ANG/MOG/MTG and the bilateral calcarine (CAL)/lingual (LING) and increased FC between the left PUT and the bilateral CER 8/9. Previous studies have identified that functional connections between the visual cortices contribute to the formation of stereovision (Freeman, 1996; Nasr et al., 2016; Abed Rabbo et al., 2018). Thus, the decreased FC between the right ANG/MOG/MTG and the bilateral CAL/LING might reflect the impaired binocular and stereoscopic vision in individuals with CE.

In this study, the SVM classification reaches a total accuracy of 93 and 90% and the AUC of 0.93 and 0.90 based on ALFF and fALFF values, respectively. Thus, ALFF and fALFF maps might be sensitive biomarkers for discriminating those groups.

Some limitations should be mentioned in this study. First, the sample size is small. Second, BOLD signals can be influenced by physiological noise, which might be a bad influence on the result of ALFF values. Third, the patients with CE showed different strabismus angles, which might be reflecting the consistency of ALFF results.

## Conclusion

Our results highlight that patient with CE had abnormal brain neural activities including MOG and supplementary motor area/PreCG, which might reflect the neural mechanism of eye movements and stereovision dysfunction in patients with CE. Moreover, ALFF and fALFF could be sensitive biomarkers for distinguishing patients with CE from HCs.

## Data Availability Statement

The raw data supporting the conclusions of this article will be made available by the authors, without undue reservation.

## Ethics Statement

The studies involving human participants were reviewed and approved by Medical Ethics Committee of the Jiangxi Provincial People's Hospital. Written informed consent to participate in this study was provided by the participants' legal guardian/next of kin.

## Author Contributions

XH, JC, and HJ contributed to data collection, statistical analyses, and wrote the manuscript. XH, JC, HJ, and Y-LZ designed the protocol and contributed to the MRI analysis. XH and JC designed this study, oversaw all clinical aspects of study conduct, and manuscript preparation. All authors contributed to the study and approved the submitted version.

## Conflict of Interest

The authors declare that the research was conducted in the absence of any commercial or financial relationships that could be construed as a potential conflict of interest.

## Publisher's Note

All claims expressed in this article are solely those of the authors and do not necessarily represent those of their affiliated organizations, or those of the publisher, the editors and the reviewers. Any product that may be evaluated in this article, or claim that may be made by its manufacturer, is not guaranteed or endorsed by the publisher.
